# Supplementary material for: Transcriptomic analysis reveals ethylene signal transduction genes involved in pistil development of pumpkin
Source: PeerJ. 2020 Aug 18;8:e9677. doi: 10.7717/peerj.9677 (PMC7442037; doi:10.7717/peerj.9677)
Supplement: Supplemental Information 1 [file peerj-08-9677-s001.docx]

***Supplementary Material***

Transcriptomic analysis reveals ethylene signal transduction genes involved in pistil development of pumpkin

Qing-Fei Li^1,2*^, Li Zhang^1,2^, Fei-Fei Pan^1,2^, Wei-Li Guo^1,2^, Bi-Hua Chen^1,2^, He-Lian Yang^1,2^, Guang-Yin Wang^1,2^, Xin-Zheng Li^1,2*^

^1^ College of Horticulture and Landscape, Henan Institute of Science and Technology, Xinxiang, Henan,453003, China；

^2^ Henan Province Engineering Research Center of Horticultural Plant Resource Utilization and Germplasm Enhancement, Xinxiang, Henan 453003, China

**^*^ Correspondence:**

Xin-Zheng Li, Qing-Fei Li

Email address: liuzhw@hist.edu.cn; lqf1988@hist.edu.cn

**Supplementary Table 1. Primers used in this study.**

| Primer | Primer Sequence（5’-3’） |
| --- | --- |
| *ACTIN-F*  *ACTIN-R* | AGCCATCTCTCATCGGTAT  CATGGTTGAACCACCACTG |
| *AUX-IAA-F*  *AUX-IAA-R* | ATTCCAATCTCGCACTT  CTCAGCCTCTTTACATCC |
| *ARF-F*  *ARF-R* | CTGTCGCTTGTTCGGGATC  TGGCTGGTCTGAGTTCTGC |
| *TF-F*  *TF-R* | CGGGCAAGATTCTCCAT  CACGGTTCCAACGACAC |
| *ETR-F*  *ETR-R* | GCCTTGAACTATCAGATACCA  CAGGAACTCGCACAGCA |
| *TCH4-F*  *TCH4-R* | AACGGCGACCTTCTTAC  GGAGTTTCCAGGCACAA |
| *CYCD3-F*  *CYCD3-R* | CGAGACGGCATTGTTAC  TTTGGTCCTTTGGTTGG |
| *JAR1-F*  *JAR1-R* | AAGCACGATGAAGACGA  TACAAAGATTGGTGGAAGT |
| *NPR1-F*  *NPR1-R* | AACGCATCATCCACAGC  AGATAAACCAACCTCGTGTC |
| *TGA-F*  *TGA-R* | CAGCCATCGTCAAACCA  ACCCATAACGCCACCAC |
| *ERDBF3-F*  *ERDBF3-R* | TTGTCACTCCAAGCGATGT  AGGGTCCTCAAAGTTCAAGAT |
| *ERTF10-F*  *ERTF10-R* | AAGCGGCAAGAGCGTAT  GGAGTCCGACAGCATTTC |
| *AP23100-F*  *AP23100-R* | GAAGATTAGTGAGCGGATGA  CCGTTGCTGCCTCGTGT |
| *ACO-F*  *ACO-R* | CCGACGAATACAGAGCC  CTCGCAAAGTAAATCCAAA |
| *PAL-F*  *PAL-R* | CGCTCCCTTCCAACAAC  TCAGCCAACTCGACCAT |
