# Supplementary material for: Transcriptomic analysis reveals ethylene signal transduction genes involved in pistil development of pumpkin
Source: PeerJ. 2020 Aug 18;8:e9677. doi: 10.7717/peerj.9677 (PMC7442037; doi:10.7717/peerj.9677)
Supplement: Supplemental Information 4 [file peerj-08-9677-s004.docx]

***Supplementary Material***

Transcriptomic analysis reveals ethylene signal transduction genes involved in pistil development of pumpkin

Qing-Fei Li^1,2*^, Li Zhang^1,2^, Fei-Fei Pan^1,2^, Wei-Li Guo^1,2^, Bi-Hua Chen^1,2^, He-Lian Yang^1,2^, Guang-Yin Wang^1,2^, Xin-Zheng Li^1,2*^

^1^ College of Horticulture and Landscape, Henan Institute of Science and Technology, Xinxiang, Henan,453003, China；

^2^ Henan Province Engineering Research Center of Horticultural Plant Resource Utilization and Germplasm Enhancement, Xinxiang, Henan 453003, China

**^*^ Correspondence:**

Xin-Zheng Li, Qing-Fei Li

Email address: liuzhw@hist.edu.cn; lqf1988@hist.edu.cn

**Supplementary Table 4. List of DEGs annotated as predicted ethylene related genes in NCBI blast** **and *Cucurbita moschata* genome.**

| Gene ID | Gene annotation | Fold change | FDR |
| --- | --- | --- | --- |
| CmoCh01G000430 | PREDICTED: AP2-like ethylene-responsive transcription factor AIL1 [*Cucumis melo*] LOC103501989 | 0.100348376 | 0 |
| CmoCh01G012750 | PREDICTED: ethylene-responsive transcription factor 4 [*Cucumis melo*] LOC 103501853 | 0.366574741 | 1.37E-126 |
| CmoCh01G014410 | PREDICTED: ethylene-responsive transcription factor ERF014 [*Cucumis melo*] LOC 103487575 | 2.785753423 | 6.54E-10 |
| CmoCh01G014870 | PREDICTED: ethylene-responsive transcription factor ERF043-like [*Cucumis sativus*] LOC101217333 | 0.113426508 | 7.98E-54 |
| CmoCh01G015350 | PREDICTED: ethylene-responsive transcription factor ERF003-like [*Cucumis sativus*] LOC101207788 | 0.439562352 | 3.20E-05 |
| CmoCh01G017440 | PREDICTED: ethylene-responsive transcription factor ERF017-like [*Cucumis melo*] LOC103493713 | 0.47163128 | 1.85 E-03 |
| CmoCh02G006870 | PREDICTED: ethylene-responsive transcription factor ERF069-like [*Cucumis melo*] LOC103496254 | 5.587529898 | 5.83E-10 |
| CmoCh02G009910 | PREDICTED: ethylene-responsive transcription factor ERF105 [*Cucumis melo*] LOC103482544 | 0.44093865 | 3.01E-27 |
| CmoCh02G011250 | PREDICTED: ethylene-responsive transcription factor CRF1-like [*Cucumis melo*] LOC103482947 | 0.008092928 | 1.11E-13 |
| CmoCh02G017950 | PREDICTED: ethylene-responsive transcription factor 1B-like [*Cucumis melo*] LOC103489880 | 0.06127232 | 4.73 E-02 |
| CmoCh03G002510 | PREDICTED: ethylene-responsive transcription factor ERN1 [*Cucumis sativus*] LOC101203179 | 0.241248375 | 3.39E-05 |
| CmoCh03G008360 | PREDICTED: ethylene-responsive transcription factor ERF060 [*Cucumis melo*] LOC103484983 | 0.053419291 | 1.31E-248 |
| CmoCh03G009340 | PREDICTED: AP2-like ethylene-responsive transcription factor ANT isoform X1 [*Cucumis sativus*] LOC101203323 | 0.371151663 | 0 |
| CmoCh03G012770 | ethylene-responsive transcription factor 4-like [*Cucumis sativus*] LOC101222924 | 0.348754543 | 1.23E-113 |
| CmoCh04G002690 | PREDICTED: ethylene-responsive transcription factor RAP2-7 [*Cucumis melo*] LOC103488740 | 0.238445005 | 2.07E-02 |
| CmoCh04G005340 | PREDICTED: ethylene-responsive transcription factor ERF109 [*Cucumis melo*] LOC103496155 | 0.041699896 | 6.82E-03 |
| CmoCh04G007400 | PREDICTED: ethylene-responsive transcription factor ERF061-like [*Cucumis sativus*] LOC101206889 | 0.091773157 | 2.73E-91 |
| CmoCh04G007530 | PREDICTED: ethylene-responsive transcription factor CRF2-like [*Cucumis melo*] LOC103493117 | 8.534829687 | 3.44E-44 |
| CmoCh04G008690 | PREDICTED: ethylene insensitive 3-like 4 protein [*Ziziphus jujuba*] LOC107425425 | 0.184353126 | 3.53E-19 |
| CmoCh04G009220 | PREDICTED: AP2-like ethylene-responsive transcription factor AIL6 [*Cucumis melo*] LOC103493271 | 0.241248375 | 3.39E-05 |
| CmoCh04G013100 | PREDICTED: ethylene-responsive transcription factor RAP2-7 [*Cucumis melo*] LOC103488740 | 7.721453081 | 2.06E-12 |
| CmoCh04G018620 | PREDICTED: ethylene-responsive transcription factor ESR2-like [*Cucumis melo*] LOC103488969 | 0.011728388 | 1.46E-09 |
| CmoCh04G024610 | PREDICTED: ethylene-responsive transcription factor ERF014-like [*Cucumis melo*] LOC103503222 | 0.336360755 | 7.04E-06 |
| CmoCh04G025710 | PREDICTED: ethylene-responsive transcription factor ERF113-like [*Cucumis melo*] LOC103485606 | 17.74247277 | 4.73E-02 |
| CmoCh05G000510 | PREDICTED: ethylene-responsive transcription factor ESR1 [*Cucumis melo*] LOC103487721 | 0.012886114 | 1.11E-08 |
| CmoCh05G001250 | PREDICTED: AP2-like ethylene-responsive transcription factor ANT [*Cucumis melo*] LOC103487813 | 0.192547857 | 0 |
| CmoCh05G001680 | PREDICTED: ethylene-responsive transcription factor RAP2-1 [*Cucumis melo*] LOC103487870 | 76.34112748 | 2.18E-08 |
| CmoCh05G003620 | PREDICTED: ethylene-responsive transcription factor ERF003-like [*Cucumis melo*] LOC103488338 | 0.229963609 | 1.15E-46 |
| CmoCh06G008250 | PREDICTED: ethylene-responsive transcription factor 5 [*Cucumis melo*] LOC103490863 | 0.385921005 | 3.12E-13 |
| CmoCh06G008260 | ethylene responsive transcription factor 1a [*Citrullus lanatus* subsp. vulgaris] AB490000.1 | 0.428495348 | 5.01E-29 |
| CmoCh06G008950 | PREDICTED: ethylene-responsive transcription factor 3 [*Cucumis melo*] LOC103491089 | 0.433318761 | 2.27E-06 |
| CmoCh07G002230 | PREDICTED: ethylene-responsive transcription factor 4-like [*Cucumis melo*] LOC103484503 | 0.421932104 | 6.89E-39 |
| CmoCh07G005390 | PREDICTED: ethylene-responsive transcription factor ERF056 [*Cucumis sativus*] LOC101211347 | 2.166582006 | 3.87E-03 |
| CmoCh08G001530 | PREDICTED: ethylene-responsive transcription factor ERF039-like [*Cucumis melo*] LOC103499073 | 2.557932492 | 1.09E-09 |
| CmoCh08G002930 | PREDICTED: ethylene-responsive transcription factor ERF098-like [*Cucumis melo*] LOC103492603 | 0.483532856 | 8.83E-04 |
| CmoCh08G004320 | ethylene receptor 1 [*Cucurbita pepo*] JX014245.1 | 0.482161155 | 1.28E-10 |
| CmoCh08G005880 | PREDICTED: ethylene-responsive transcription factor ERF003-like [*Cucumis melo*] LOC103499832 | 2.666504733 | 4.58E-11 |
| CmoCh09G000820 | PREDICTED: 24-methylenesterol C-methyltransferase 2 [*Cucumis melo*] LOC103503852 | 0.452938188 | 6.16E-27 |
| CmoCh09G003100 | PREDICTED: AP2-like ethylene-responsive transcription factor AIL1 isoform X1 [*Cucumis melo*] LOC103493625 | 0.349331792 | 0 |
| CmoCh09G008400 | PREDICTED: ethylene-responsive transcription factor 4 [*Cucumis melo*] LOC103501853 | 0.373759512 | 1.28E-09 |
| CmoCh09G013360 | PREDICTED: carboxymethylenebutenolidase homolog [*Citrus sinensis*] LOC102613358 | 0.422019634 | 1.12E-73 |
| CmoCh10G008120 | PREDICTED: ethylene-responsive transcription factor ERF017-like [*Cucumis melo*] LOC103492391 | 0.396562266 | 5.05E-05 |
| CmoCh11G005450 | PREDICTED: ethylene-responsive transcription factor WIN1-like [*Cucumis melo*] LOC103487449 | 0.222613016 | 7.04E-27 |
| CmoCh11G007650 | PREDICTED: ethylene-responsive transcription factor ERF017-like [*Cucumis melo*] LOC103492391 | 0.079509726 | 3.19E-17 |
| CmoCh11G019920 | PREDICTED: ethylene-responsive transcription factor 4 [*Cucumis melo*] LOC103495486 | 0.390762367 | 1.43E-29 |
| CmoCh12G005500 | AP2-like ethylene-responsive transcription factor ANT [*Cucumis melo*] LOC103487813 | 0.422438693 | 9.47E-36 |
| CmoCh12G012980 | PREDICTED: ethylene-responsive transcription factor ERF039 [*Cucumis sativus*] LOC101212945 | 2.368451567 | 9.44E-03 |
| CmoCh13G006500 | PREDICTED: ethylene-responsive transcription factor 12 [*Cucumis melo*] LOC103486573 | 0.378903764 | 3.67E-24 |
| CmoCh14G016730 | PREDICTED: ethylene-responsive transcription factor ERF086 [*Cucumis melo*] LOC103483813 | 2.7097037 | 3.15E-06 |
| CmoCh14G020660 | PREDICTED: ethylene-responsive transcription factor ERF071-like [*Cucumis melo*] LOC103483310 | 0.403030492 | 4.51E-09 |
| CmoCh14G022160 | PREDICTED: ethylene-responsive transcription factor ERF038-like [*Cucumis melo*] LOC103483130 | 0.48816761 | 5.24E-04 |
| CmoCh15G003420 | PREDICTED: 2-methylene-furan-3-one reductase-like [*Cucumis melo*] LOC103498067 | 0.484599755 | 9.67E-268 |
| CmoCh15G005930 | PREDICTED: ethylene-responsive transcription factor ERF113-like [*Cucumis melo*] LOC103485606 | 2.623684775 | 2.96E-04 |
| CmoCh15G012620 | PREDICTED: ethylene-responsive transcription factor ERF027 [*Cucumis melo*] LOC103482708 | 0.025444349 | 1.32 E-04 |
| CmoCh15G013340 | PREDICTED: ethylene-responsive transcription factor CRF2-like [*Cucumis melo*] LOC103482804 | 0.16391171 | 1.60E-06 |
| CmoCh15G014020 | PREDICTED: ethylene-responsive transcription factor ERF096-like [*Cucumis melo*] LOC103482887 | 0.06127232 | 4.73E-02 |
| CmoCh15G014220 | PREDICTED: ethylene-responsive transcription factor ERF105 [*Cucumis melo*] LOC103482544 | 0.360572497 | 1.19E-53 |
| CmoCh16G006010 | PREDICTED: ethylene-responsive transcription factor ERF061-like [*Cucumis sativus*] LOC101206889 | 0.253805831 | 9.66E-49 |
| CmoCh16G007500 | PREDICTED: ethylene insensitive 3-like 4 protein [*Ziziphus jujuba*] LOC107425429 | 0.031604397 | 9.59E-04 |
| CmoCh16G008040 | PREDICTED: AP2-like ethylene-responsive transcription factor AIL6 [*Cucumis melo*] LOC103493271 | 0.304566795 | 1.29E-22 |
| CmoCh16G011670 | ethylene responsive transcription factor 1a [*Citrullus lanatus* subsp. vulgaris] AB490000.1 | 0.386487681 | 1.51E-04 |
| CmoCh17G013120 | PREDICTED: ethylene-responsive transcription factor ERF098-like [*Cucumis melo*] LOC103492603 | 2.062467479 | 2.20E-03 |
| CmoCh17G013460 | PREDICTED: ethylene-responsive transcription factor ERF113 [*Cucumis sativus*] LOC101205898 | 0.09598464 | 1.36E-13 |
| CmoCh18G010350 | PREDICTED: ethylene-responsive transcription factor ERF118 [*Cucumis melo*] LOC103489279 | 0.424235171 | 3.08E-10 |
| CmoCh18G013230 | PREDICTED: ethylene-responsive transcription factor CRF4-like [*Cucumis melo*] LOC103484149 | 2.081649205 | 2.02E-34 |
| CmoCh19G000970 | PREDICTED: ethylene-responsive transcription factor 1B-like [*Cucumis melo*] LOC103489880 | 0.021293925 | 3.50E-05 |
| CmoCh19G001880 | PREDICTED: ethylene-responsive transcription factor-like protein At4g13040 isoform X1 [*Cucumis melo*] LOC103499216 | 0.440773801 | 7.55E-04 |
| CmoCh19G010170 | PREDICTED: ethylene-responsive transcription factor ERF054 [*Cucumis melo*] LOC103495520 | 34.48494555 | 9.59E-04 |
| CmoCh19G010380 | PREDICTED: ethylene-responsive transcription factor 4 [*Cucumis melo*] LOC103495486 | 0.476053204 | 4.67E-40 |
| CmoCh20G011330 | PREDICTED: ethylene-responsive transcription factor ERF110-like [*Cucumis melo*] LOC103497485 | 0.06127232 | 4.73E-02 |
