# Supplementary material for: Transcriptomic analysis reveals ethylene signal transduction genes involved in pistil development of pumpkin
Source: PeerJ. 2020 Aug 18;8:e9677. doi: 10.7717/peerj.9677 (PMC7442037; doi:10.7717/peerj.9677)
Supplement: Supplemental Information 6 [file peerj-08-9677-s006.docx]

***Supplementary Material***

Transcriptomic analysis reveals ethylene signal transduction genes involved in pistil development of pumpkin

Qing-Fei Li^1,2*^, Li Zhang^1,2^, Fei-Fei Pan^1,2^, Wei-Li Guo^1,2^, Bi-Hua Chen^1,2^, He-Lian Yang^1,2^, Guang-Yin Wang^1,2^, Xin-Zheng Li^1,2*^

^1^ College of Horticulture and Landscape, Henan Institute of Science and Technology, Xinxiang, Henan,453003, China；

^2^ Henan Province Engineering Research Center of Horticultural Plant Resource Utilization and Germplasm Enhancement, Xinxiang, Henan 453003, China

**^*^ Correspondence:**

Xin-Zheng Li, Qing-Fei Li

Email address: liuzhw@hist.edu.cn; lqf1988@hist.edu.cn


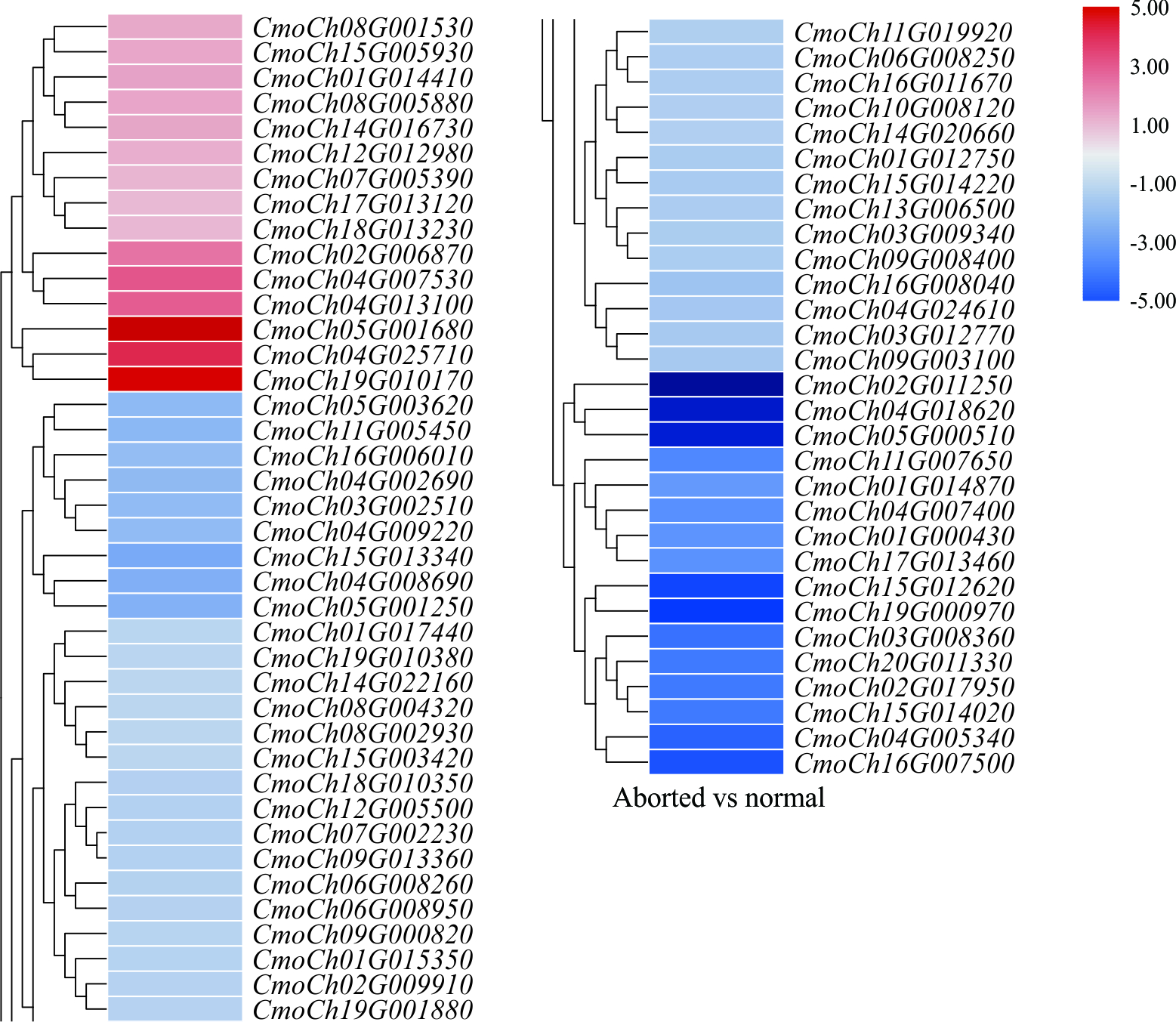


**Supplementary Figure 1. Heatmap visualization of predicted ethylene related DEGs.**
